# Chronic urticaria following COVID-19 mRNA vaccines

**DOI:** 10.1016/j.jdcr.2024.04.041

**Published:** 2024-05-07

**Authors:** Öner Özdemir

**Affiliations:** Division of Allergy and Immunology, Department of Pediatrics, Research and Training Hospital of Sakarya University, Sakarya University Medical Faculty, Adapazarı, Sakarya, Türkiye

**Keywords:** chronic urticaria, COVID-19, vaccine

*To the Editor:* I have read the article titled “Chronic urticaria after Moderna COVID-19 vaccine boosters: A case series” by Ryan et al.^1^ with great interest. When I read this article about the COVID-19 vaccine, I thought a few things should be clarified, as it is in my area of interest.

First, when describing chronic urticaria (CU) in the introduction of the case report,^1^ it was slightly confused with recurrent urticaria. Namely, CU is, of course, recurrent, but in addition to chronic spontaneous and inducible urticaria, vasculitic urticaria, autoinflammatory urticaria (NLRP12-associated cold-induced autoinflammatory syndrome), etc. can be included in the recurrent urticaria class.^2^ However, what is meant here is simply chronic spontaneous urticaria. It is actually important for the authors to explain exactly what they mean here.

Second, there are contradictory statements in the article from the point of view of an allergist/immunologist. In one place, it mentions that the CU that develops after vaccination may be due to a higher concentration of messenger RNA (mRNA), while in another place, it mentions another unknown cause.^1^ Comirnaty vaccine contains 30 micrograms per dose, and Moderna contains 50 micrograms per dose of mRNA.^3^ The fact that urticaria with one does not occur with the slightly lower dose in the other seems to be far from being the real cause.

When I examined the official European Medicines Agency website in detail, I saw a somewhat important difference in terms of allergenicity between Moderna and Comirnaty vaccines. Moderna, which causes CU, contains polyethylene glycol (PEG) 2000-DMG (1,2-dimyristoyl-rac-glycero-3-methoxypolyethylene glycol-2000), SM-102, Trometamol,^4^^,^^5^ and the other does have just PEG2000. PEG is known to be responsible for many other allergic reactions such as anaphylaxis.^6^^,^^7^ Tromethamine or trometamol and other excipients in mRNA vaccines are discussed in detail in a recent article in terms of their potential allergenicity.^5^^,^^8^

As far as can be understood from the table,^1^ even though it is said that anaphylaxis did not occur, symptoms suggestive of anaphylaxis were actually observed in 2 cases. This is consistent with the allergenic excipients including PEG content of the vaccine.^4^, ^5^, ^6^, ^7^

Third, in the table,^1^ BNT162b2 and Comirnaty vaccines are shown as 2 separate vaccines when writing the vaccines for case 1. They are the same vaccine. However, as is known, the code name is BNT162b2, and the registered name is Comirnaty. Also in the table, Comimaty is written/misspelled instead of the trademark Comirnaty (Pfizer).^1^

Fourth, although these case series suggest that CU may be caused by a particular mRNA vaccine (Moderna), there are ample reports in the literature that CU may occur with inactivated, vector, and other types of mRNA vaccines.^9^ We even have our own cases and experiences with inactive vaccines.^10^

The occurrence of urticaria in patients with dermatographism (chronic inducible urticaria) may suggest that urticaria is triggered by the pathophysiology of the vaccines that cause urticaria in these patients. Indeed, this article does not discuss whether these vaccines themselves initiate CU (new-onset) or exacerbate urticaria in the setting of existing CU such as dermatographism.^10^ This case series actually includes examples of both conditions.

In conclusion, I would like to thank the authors for this nice case series, its results, and their comments. This study is valuable in showing that mRNA vaccines, especially the Moderna vaccine, cause CU. In addition, solving the problem by changing the mRNA vaccine product is also important for patient care.

## Conflicts of interest

None disclosed.
